# A spinal and bulbar muscular atrophy (SBMA) disease-specific human embryonic stem cell (hESC) line, UMICHe002-A/UM197–1

**DOI:** 10.1016/j.scr.2024.103548

**Published:** 2024-09-07

**Authors:** Indri Erliandri, Agamjot Sangotra, Laura Keller, Andrew P. Lieberman, Gary D. Smith

**Affiliations:** a Departments of Obstetrics and Gynecology, Physiology, and Urology, University of Michigan, Ann Arbor, MI, United States; b MStem Cell Laboratories, University of Michigan, Ann Arbor, MI, United States; c Department of Pathology, University of Michigan, Ann Arbor, MI, United States

## Abstract

Spinal and Bulbar Muscular Atrophy (SBMA) is an X-linked degenerative disorder of the neuromuscular system that is caused by an expanded CAG/polyglutamine (polyQ) tract within the *Androgen Receptor* (*AR*) gene. This mutation causes progressive muscle weakness and atrophy in men. Here, we report the establishment of the first SBMA disease-specific human embryonic stem cell (hESC) line in the NIH hESC registry, UM197–1. UM197–1 exhibits pluripotency, the ability to differentiate into three germ layers *in vitro*, and provides a new cellular model system to study SBMA disease pathogenesis.

## 1. Resource Table

**Table:** 

Unique stem cell line identifier	UMICHe002-A
Alternative name(s) of stem cell line	UM197–1; SBMA hESC
Institution	MStem Cell Laboratories, University of Michigan
Contact information of distributor	Gary D. Smith, smithgd@med.umich.edu
Type of cell line	ESC
Origin	Human
Additional origin info	Sex: Male
Cell Source	Abnormal blastocyst with expanded CAG repeat mutation in the AR gene
Clonality	Mixed
Method of reprogramming	N/A
Genetic Modification	NO
Type of Modification	N/A
Evidence of the reprogramming transgene loss	N/A
Associated disease	Spinal and bulbar muscular atrophy (SBMA) (OMIM: 313200)
Gene/locus	Hemizygous pathogenic CAG repeat expansion in AR (repeat length = 49 CAGs) (HGNC: 644)
Date archived/stock date	05/29/2019
Cell line repository/bank	NIH Human Embryonic Stem Cell Registry (#0399) https://grants.nih.gov/stem_cells/ registry/current.htm?ID=NIHhESC-19–0399
Unique stem cell line identifier	UMICHe002-A
Ethical approval	University of Michigan, Human Pluripotent Stem Cell Research Oversight (HPSCRO) Program, #1084 IRB Approval - HUM00028742

## 2. Resource Utility

There remains an incomplete understanding of the pathogenic cascade underlying neuromuscular degeneration in SBMA. UM197–1 hESC harbors a CAG repeat expansion in the endogenous *AR* locus within the range that occurs in SBMA patients. This resource provides a new experimental model system in which to explore disease mechanisms.

## 3. Resource Details

**Fig. 1.**
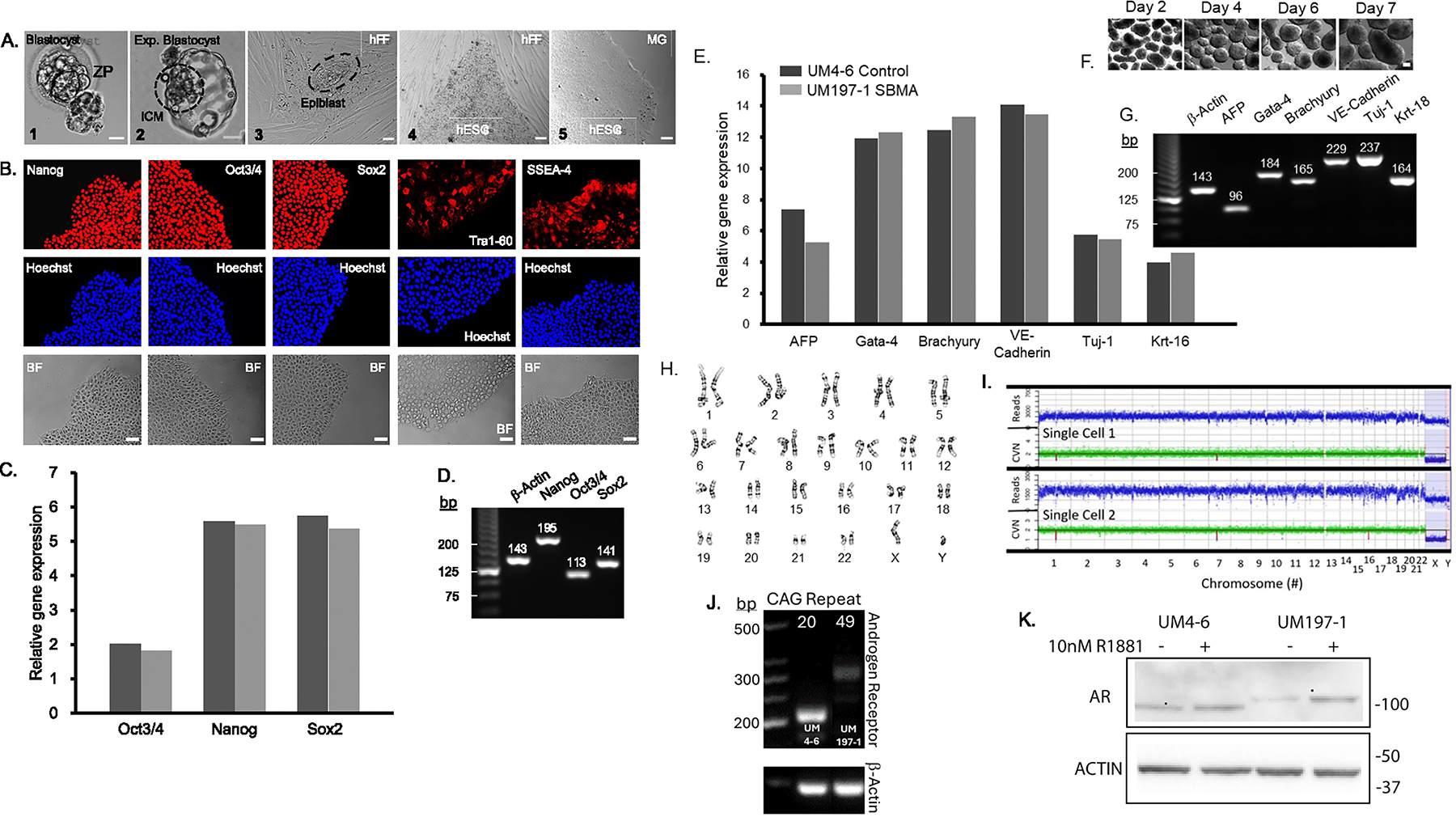


UM197–1 hESC line was generated from a donated human embryo identified as carrying a hemizygous CAG repeat expansion in the *AR* gene by preimplantation genetic testing for monogenetic disease (PGT-M). UM197–1 was negative for Mycoplasma contamination. Short tandem repeat (STR) analysis examining fifteen STR loci plus Amelogenin was performed on passage 13 UM197–1 hESC. Cell line DNA fingerprinting results demonstrated a STR profile consistent with a single human cell line not matching any profile published in the ATCC, NIH, or DSMZ websites. Generated UM197–1 hESC displayed a normal morphology (Fig. 1A) and expressed pluripotency markers SOX2, NANOG, OCT3/4, TRA 1–60, and SSSEA-4 as demonstrated through qualitative immunocytochemical analysis (Fig. 1B). Quantitative real-time PCR revealed expression of pluripotency markers *OCT3/4*, *NANOG,* and *SOX2* (Fig. 1C, D). Functional pluripotency of UM197–1 was demonstrated by gene expression of lineage markers AFP, GATA-4 (endoderm), BRACHYURY, VE-Cadherin(mesoderm), and TUJ-1, Krt-18 (ectoderm) in day 7 embryoid bodies differentiated from UM197–1 hESC (Fig. 1E, F, G). G-banded cytogenetic analysis of UM197–1 hESC identified a normal 46XY karyotype with no chromosomal abnormalities (Fig. 1H), as confirmed by evaluation of multiple single cells, whole genome amplification, and next gen sequencing copy number variance (CNV; Fig. 1I). Fragment analysis PCR identified an *AR* CAG repeat length of 49 (Fig. 1J). Western blot analysis revealed AR protein expression in UM197–1 hESC (Fig. 1K). In addition, the polyQ AR protein migrates more slowly on Western than the WT protein and ligand-dependent (AR-agonist R1881) stabilization occurs with both WT and polyQ AR. These effects are similar to prior reports (Lieberman et al., 2002) and hence validate the model system. These features are summarized in Table 1.

## 4. Materials and methods

### 4.1. Isolation and cell culture

Human embryos were originally created by assisted reproductive technologies for the purpose of procreation and tested with preimplantation genetic testing (PGT) to identify embryos with CAG repeat expansions within exon 1 of the *AR* gene (chromosome Xq12) at an independent fertility healthcare provided. The embryos with CAG repeat expansions were donated to the University of Michigan under MStem Cell Laboratory’s Institutional Review Board (IRB) approved study “derivation of human Embryonic Stem Cells” (HUM00028742). Written informed consent was obtained for all embryo donations.

Derivation of hESC, derivative expansion, and characterizations were performed with non-federal funds prior to acceptance to the NIH registry. The day-5 (day 0 – day of fertilization) SBMA-affected (X,Y) embryo was cryopreserved, shipped to the University of Michigan, thawed, and incubated in G2+ media (Vitrolife) at 37 °C, 5 % CO_2_, 5 % O_2_ in air for 24 h. The expanded/hatched embryo was removed from the zona pellucida and biopsied using a microscope and laser at 300–400 μS pulse. The laser-isolated Inner Cell Mass (ICM) was cultured on human foreskin fibroblast (HFF)-irradiated feeders in Xeno-Free (XF-Knock-out DMEM-Gibco, #1029) media supplemented with 20 ng basic fibroblast growth factor-2 (bFGF-2-MilliporeSigma, #GF003AF-100UG), 1 mM Glutamax (Gibco, #35050061), 0.1 mM 2-mercaptoethanol (Aldrich, #M6250), 1 % Non-essential Amino Acids (Gibco, #11140050) and XF-knock-out serum replacement (XF-KOSR-Gibco, #12618012)) as a protein source.

The plated ICM gave rise to an epiblast-like structure that was manually dissected into two pieces and passaged into new dishes on HFFs in XF supplemented media (Passage 1; P1). Following derivation hESC colonies were maintained and expanded until P7 on HFFs feeder in XF supplemented media (as above), incubated at 37 °C, 5 % CO_2_, and 5 %O_2_ in air. Manual passaging of hESC colonies was performed by cutting colonies with a glass blade and transferring multiple ~0.2 cm hESC clumps to new HFFs and supplemented media using a micro-pipette. At P8 onward, colonies were transferred and maintained in mTeSR1 media (Stem Cell Technologies) containing bFGF-2 and KOSR on Matrigel (MG; Corning, #354277)-coated plates with daily media changes, incubated at 37 °C, 5 % CO_2_ in air, and passaged manually with using L7 passaging solution (Lonza, #FP-5013). Cryopreservation of hESCs were performed before P8 (freezebacks on HFFs) and early after P8 (freezebacks on MG).

### 4.2. Mycoplasma screening

Mycoplasma contamination was tested as previously described (Moore et al., 2022).

### 4.3. Immunocytochemistry and imaging

Immunocytochemical analysis was performed on UM197–1 hESC with antibodies against pluripotency markers SOX2, NANOG, OCT3/4, TRA 1–60, and SSSEA-4 (Table 2). Cell nuclei were labelled with Hoechst (ThermoFisher Scientific, #33258) and imaged using an Olympus, model IX71 fluorescence microscope (Fig. 1B). Colony morphology was simultaneously assessed by phase-contrast brightfield microscopy (Fig. 1B).

### 4.4. Embryoid body formation and analysis

At passage 7 UM197–1 hESCs were cultured on a feeder-free matrix (Matrigel, Corning # 354277) in mTeSR1 media (StemCell Technologies, #85850) until ~ 80 % confluency and then harvested mechanically and used to form EBs for lineage marker detection by quantitative real-time PCR in triplicate as previously described (Moore et al., 2022; Table 2, Fig. 1E).

### 4.5. Karyotyping

G-banding was performed on 20 metaphase spreads of UM197–1 cells at passage 13 by Cell Line Genetics (Madison, WI). Metaphase spreads were evaluated at 100X with a Leica GSL Scanner (100X objective, Leica GSL 120 CytoVision, Leica MicroSystems, Buffalo Grove, IL) with ban –count resolution of ~475. Cytogenic analysis demonstrated an apparently normal male 46, XY karyotype in 19 cells with 1 cell demonstrated non-clonal aberrations ruled as most likely artefacts of culture. UM197–1 hESC karyotype of 46XY was also evaluated and confirmed by in-house multi-cell single cell DNA Sequencing following whole genome amplification and library preparation (ResolveOME Kit; BioSkryb1#00500; University of Michigan Advanced Genomics Core), and ~2.5 % coverage next gen sequencing with NovaSeq S4–300 yielding CNV.

### 4.6. STR analysis

Cell Line DNA Fingerprinting was performed on UM197–1 passage 13 (Cell Line Genetics, Madison, Wisconsin) as previously defined (Moore et al., 2022) and presented in addendum 1.

### 4.7. Sequencing analysis

For CAG repeat size determination, total DNA was extracted using GenJet Genomic DNA purification kit (Thermo Scientific, K0721) according to the manufacturer’s protocol. PCR was performed using *AR* primers (Table 2). Purified PCR product was visualized in 1 % agarose gel and sequenced (Laragen).

### 4.8. Protein Isolation and Western blot

hESCs were collected at p20 and washed in 1x PBS. Cell pellets were lysed in RIPA buffer (Teknova) containing complete Mini Protease Inhibitor Cocktail (Roche). Following sonication, samples were spun down at 15,000 *g* for 15 min and protein concentration of the supernatant was calculated using DC assay (Bio-Rad). Equal amounts of protein were loaded into NuPAGE 4–12 %, 12 + 2-well Midi gels (Invitrogen, WG1401BX10) and run in 1 × MOPS buffer (Invitrogen, NP0001). The gels were transferred to PVDF membranes using a Semi-Dry Transfer System (Bio-Rad). Blots were blocked for 30 min in 5 % nonfat dry milk in TBS containing 0.1 % Tween and placed into primary solution (1:1,000 Milipore PG-21, 1:2,000 Invitrogen Actin) diluted in blocking solution at 4 °C overnight. Goat anti-rabbit HRP (Bio-Rad) and goat anti-mouse HRP (Bio-Rad) were diluted at 1:2,000 in 5 % nonfat dried milk and incubated for 1 h at room temperature. Immunoreactivity was detected with SuperSignal West Pico PLUS Chemiluminescent Substrate (Thermo Scientific 34577) via an iBright FL1500 imaging system (Invitrogen A44241).

## Figures and Tables

**Table 1:** Characterization and validation.

Classification	Test	Result	Data

Morphology	Photography Bright field	Normal	Fig. 1A
Phenotype	Qualitative analysis: Immunocytochemical	Positive for pluripotency markers: SOX2, NANOG, OCT3/4, TRA 1–60, and SSSEA-4	Fig. 1B
	Quantitative analysis: RT-qPCR	Positive for pluripotency markers: *OCT3/4, NANOG*, and *SOX2*	Fig. 1C, D
Genotype	Karyotype (G-banding) and resolution	46XY, band-count resolution ~ 475	Fig. 1H
	Karyotype (copy number variant) multi-cell single cell DNASeq	46XY	Fig. 1I
Identity	Microsatellite PCR	Not performed	N/A
Mutation analysis	STR analysis	15 STR loci in passage 18 cells. Result: human with no match found in ATCC, NIH, or DSMZ databases.	Submitted in archive with journal
	Gene fragment analysis for AR CAG repeat length	Hemizygous pathogenic CAG repeat expansion in AR. AR CAG repeat = 49	N/A
Microbiology and virology	Mycoplasma	Mycoplasma testing by RT-PCR: Negative	N/A
Differentiation potential	Embryoid body formation	Following undirected differentiation (EB formation), PCR analysis confirmed gene expression of markers of three germ layers (endoderm: *alpha-fetoprotein (AFP)*, GATA-4; mesoderm: *BRACHYURY, VE-Cadherin*; ectoderm: TUJ-1, Krt-18).	Fig. 1E, F, G
Donor screening (OPTIONAL)	HIV 1 + 2 Hepatitis B, Hepatitis C	N/A	N/A
Genotype additional info (OPTIONAL)	Blood group genotyping	N/A	N/A
Genotype additional info (OPTIONAL)	HLA tissue typing	N/A	N/A

**Table 2:** Reagents details.

	Antibodies used for immunocytochemistry/flow-cytometry			
	Antibody	Dilution	Company Cat #	RRID

Pluripotency Markers	Rabbit anti-NANOG	1:150	Abcam ab21624	RRID: AB_44637
	Goat anti-OCT3/4	1:300	Santa Cruz Biotechnology, sc-8628	RRID: AB_653551
	Rabbit anti-SOX2	1:800	EMD Millipore, AB5603	RRID: AB_2286686
	Mouse anti-Tra1-60	1:200	Millipore, MAB4360	RRID: AB_2119183
	Mouse anti-SSEA-4	1:100	Millipore MAB4304	RRID: AB_177629
Androgen Receptor	Rabbit anti-AR	1:400	Milipore 06–680	RRID:AB_310214
Secondary antibodies	Donkey anti-Rabbit	NANOG 1:200 SOX2 1:800	Jackson ImmunoResearch, 711–165–152	RRID: AB_2307443
	Donkey anti-Goat	1:800	Jackson ImmunoResearch, 705–096–147	RRID: AB_2340402
	Donkey anti-Mouse	TRA 1–60: 1:100 SSEA4 1:800	Jackson ImmunoResearch, 715–165–150	RRID: AB_2340813
	Goat anti-Rabbit	AR 1:2000	Bio-Rad 1,706,515	RRID: AB_11125142
	Goat anti-Mouse	Actin 1:2000	Bio-Rad 1,706,516	RRID: AB_2921252

Primers Marker	Target Gene	Size of band	Forward	Reverse

Pluripotency	Oct3/4	195 bp	5′-GATGGCGTACTGTGGGCCC-3′	5′-TGGGACTCCTCCGGGTTTTG-3′
	Nanog	113 bp	5′-TCCTCCTCTTCCTCTATACTAAC-3′	5′-CCCACAAATCACAGGCATAG-3′
	Sox2	141 bp	5′-GAGAGAAAGAAAGGGAGAGAAG-3′	5′-GAGAGAGGCAAACTGGAATC-3′
Differentiation	AFP	96 bp	5′-AAACTATTGGCCTGTGGCGA-3′	5′-GGCCAACACCAGGGTTTACT-3′
	Gata-4	184 bp	5′-CAGATGCCTTTACACGCTGA-3′	5′-TCCGCTTGTTCTCAGATCCT-3′
	Brachyury	165 bp	5′-ACCCAGTTCATAGCGGTGAC-3′	5′- GGATTGGGAGTACCCAGGTT-3′
	VE-Cadherin	229 bp	5′-CCTACCAGCCCAAAGTGTGT-3′	5′-GAGATGACCACGGGTAGGAA-3′
	Tuj-1	237 bp	5′-ATGCGGGAGATCGTGCACAT-3′	5′-CCCTGAGCGGACACTGT-3′
	KRT-18	164 bp	5′- CACAGTCTGCTGAGGTTGGA-3′	5′-GAGCTGCTCCATCTGTAGGG-3′
Internal control	β-Actin	143 bp	5′-GCCGAGGACTTTGATTGC-3′	5′-GTGTGGACTTGGGAGAGG-3′
Targeted mutation analysis/sequencing	AR	~1000 bp	6 Fam-labeled forward: 5′-CCAGAATCTGTTCCAGAGCGTG-3′	5′-TGTTCCCCTGGACTCAGATG-3′
